# Subgroups of Long-Term Sick-Listed Based on Prognostic Return to Work Factors Across Diagnoses: A Cross-Sectional Latent Class Analysis

**DOI:** 10.1007/s10926-020-09928-5

**Published:** 2020-10-14

**Authors:** Martin Inge Standal, Lene Aasdahl, Chris Jensen, Vegard Stolsmo Foldal, Roger Hagen, Egil Andreas Fors, Marit Solbjør, Odin Hjemdal, Margreth Grotle, Ingebrigt Meisingset

**Affiliations:** 1 Department of Psychology, Faculty of Social and Educational Sciences, Norwegian University of Science and Technology, Trondheim, Norway; 2 Department of Public Health and Nursing, Faculty of Medicine and Health Sciences, Norwegian University of Science and Technology, Trondheim, Norway; 3 Unicare Helsefort Rehabilitation Centre, Rissa, Norway; 4 National Center for Occupational Rehabilitation, Rauland, Norway; 5 General Practice Research Unit, Department of Public Health and Nursing, Faculty of Medicine and Health Sciences, Norwegian University of Science and Technology, Trondheim, Norway; 6 Department of Physiotherapy, Faculty of Health Sciences, Oslo Metropolitan University, Oslo, Norway; 7 Department for Research of Musculoskeletal Disorders (FORMI), Oslo University Hospital, Oslo, Norway

**Keywords:** Sick leave, Return to work, Vocational rehabilitation, Common mental disorder, Pain

## Abstract

Comorbidity is common among long-term sick-listed and many prognostic factors for return to work (RTW) are shared across diagnoses. RTW interventions have small effects, possibly due to being averaged across heterogeneous samples. Identifying subgroups based on prognostic RTW factors independent of diagnoses might help stratify interventions. The aim of this study was to identify and describe subgroups of long-term sick-listed workers, independent of diagnoses, based on prognostic factors for RTW. Latent class analysis of 532 workers sick-listed for eight weeks was used to identify subgroups based on seven prognostic RTW factors (self-reported health, anxiety and depressive symptoms, pain, self-efficacy, work ability, RTW expectations) and four covariates (age, gender, education, physical work). Four classes were identified: Class 1 (45% of participants) was characterized by favorable scores on the prognostic factors; Class 2 (22%) by high anxiety and depressive symptoms, younger age and higher education; Class 3 (16%) by overall poor scores including high pain levels; Class 4 (17%) by physical work and lack of workplace adjustments. Class 2 included more individuals with a psychological diagnosis, while diagnoses were distributed more proportionate to the sample in the other classes. The identified classes illustrate common subgroups of RTW prognosis among long-term sick-listed individuals largely independent of diagnosis. These classes could in the future assist RTW services to provide appropriate type and extent of follow-up, however more research is needed to validate the class structure and examine how these classes predict outcomes and respond to interventions.

## Background

Prolonged sickness absence is costly for society and associated with adverse health outcomes and comorbidity for the individual [1]. In order to help individuals return to work (RTW) effective vocational rehabilitation interventions are required as healthcare treatment alone has little impact on work outcomes [2]. However, the results of such interventions are inconclusive [3–6]. The variation in effectiveness found in RTW interventions could partly be due to the effects being averaged across heterogeneous samples, meaning some subgroups will have no benefit or possibly even experience negative outcomes of these interventions [7]. Diagnosis is also often used as basis for recruitment into such interventions, even though diagnosis provides limited information of the complexity and interrelationship between factors associated with prognosis [8, 9]. For example, musculoskeletal and psychological disorders, the most prevalent diagnoses for loss of work days in Norway [10] and major causes of disability worldwide [11], have considerable comorbidity and several shared prognostic factors for RTW [12–16]. In addition, patterns of relapse between RTW and sick leave are common for both of these diagnostic categories [17, 18]. An alternative approach could be using known factors that influence RTW for early identification of subgroups at risk of prolonged sick leave, regardless of diagnosis.

Identifying subgroups that can be used to stratify care is challenging and has been a focus of research in some fields for many years [19], mainly in patients with musculoskeletal disorders [20–23]. Such stratification approaches have shown effective in treatment of patients with low back pain [24]. However, few studies have attempted to identify subgroups based on prognostic RTW factors independent of diagnoses. One recent study identified subgroups of unemployed sick-listed individuals based on their predicted risk of long-term sickness absence and found four groups characterized by negative RTW expectations, positive RTW expectations, mental limitations and physical limitations [25]. Such research is still lacking for those with an employment contract. As many social insurance and healthcare professionals serve varied user groups, identifying homogeneous subgroups independent of diagnosis could assist these services to channel resources towards those who may benefit the most [26].

The aim of the present study was to identify and describe subgroups of long-term sick-listed workers, independent of diagnoses, based on prognostic factors for RTW. In particular, we wanted to investigate the following research questions:

1. What characterizes subgroups of long-term sick listed workers, independent of diagnoses, based on prognostic factors?
2. How are the psychological and musculoskeletal diagnostic categories distributed within these subgroups?

## Methods

### Study Design

This cross-sectional study used data from a cohort of sick-listed workers in an ongoing randomized controlled trial [27]. All data in the present study were collected at inclusion in the trial, prior to randomization. The study was approved by the Regional Committee for Medical and Health Research Ethics in South East Norway (No: 2016/2300). Written informed consent was obtained from all participants.

### Study Setting

In Norway, employees are entitled to 12 months of full wage benefits when on sick leave. For the first 16 days of sick leave wages are paid by the employer, while the remaining year is paid for by the National Insurance Scheme through the Norwegian Labour and Welfare Administration (NAV) [28].

### Participants and Recruitment

Participants in the present study were employed workers aged 18–62 on sick leave for eight weeks the previous 6 months, with a current sick leave status of 50–100%. Eligible participants living in Trondheim, Central Norway, were invited via NAV’s electronic communication site. Data from participants included in the trial from August 2017 to October 2019 were used in the present study. In this period 4708 individuals were invited, of which 709 (15%) accepted and received a questionnaire by e-mail at eight weeks of sick leave. This questionnaire was answered by 571 (81%) of the included participants.

### Measurement Instruments

The questionnaire included questions related to sociodemographic characteristics, symptoms and health, and work-related factors. Variables were selected a priori based on a literature search of reviews on prognostic factors for RTW. Factors such as perceived health [29], symptom severity [13], and the possibility of workplace adjustments are predictors for prolonged sick leave [30, 31]. Furthermore, factors such as RTW self-efficacy [15], perceived work ability and RTW expectations have also been shown to be important for RTW [13, 32]. Common sociodemographic factors are age, education, gender, and the physical demands of one’s work [13, 16, 32]. In addition, information on participants’ current diagnosis was obtained from NAV.

#### Sociodemographic Characteristics

Sociodemographic factors included age, gender, educational level and the physical demands of the participants’ work. Age was scored as a continuous variable. Education was dichotomized as higher (completed minimum 3 years of college/university) or lower. Participants were asked how physically demanding their job was by describing their work using the categories “Mostly sedentary work”, “Work that demand that you walk a lot”, “Work where you walk and lift a lot”, “Heavy manual labour”, and “Do not know / unsure”. This variable was dichotomized (physically demanding work or not) by combining the two less demanding categories and the two more demanding categories. “Do not know / unsure” was set to missing (*n* = 18)*.*

#### Symptoms and Health

Anxiety was assessed using the Generalized Anxiety Disorder-7 questionnaire [33], and depression with the Patient Health Questionnaire-9 [34]. Anxiety and depression scores were combined into the Patient Health Questionnaire Anxiety and Depression Scale (PHQ-ADS), which has shown to be a valid and reliable composite measure of depression and anxiety [35]. The PHQ-ADS was used to assess anxiety and depression symptoms on a scale from 0 to 48, where 0 indicate low levels of symptoms and 48 indicate high levels of symptoms.

Pain intensity was assessed by an item from the Brief Pain Inventory [36, 37] querying participants to “Describe your average pain intensity the last week” on a scale from 0 (no pain at all) to 10 (worst possible pain).

To detect individuals who may have had other health issues besides anxiety, depression or pain, we included the EQ-VAS analog scale from the EQ-5D-5L questionnaire [38]. This question asks participants to rate their current health on a scale from 0 to 100 (0 being worst possible health and 100 being best possible health) and was used to assess general health status.

#### Work Related Factors

Workplace adjustment latitude was examined with the question “To what degree do you feel your workplace facilitates work adjustments?”. Response options ranged from 1 (to a very low degree) to 10 (to a very high degree).

Self-reported work ability was measured using the work ability score (WAS), which is an item from the Work Ability Index [39]. WAS asks participants about their “current work ability compared with lifetime best” on a scale from 0 (completely unable to work) to 10 (work ability at its best). WAS has been shown to be a good alternative to using the full index [40, 41].

Work related self-efficacy was measured using an 11-item RTW-SE scale [42]. The scale has 11 questions on expectations of working if the participants were to imagine being back to work tomorrow. The scale ranges from 0 “totally disagree to 5 “totally agree”. An average score of the 11 items was used.

Return-to-work expectations was measured by the question “Starting today, how many months do you believe you will be sick-listed?”. Answers greater than 12 months (*n* = 14) were set to 12 months, as individuals need to apply for more long-term benefits after 12 months [43].

#### Diagnosis

Diagnosis was retrieved from the sick leave certificate and obtained from NAV. Diagnosis is usually set by the individual’s general practitioner, using the International Classification of Primary Care (ICPC-2) [44]. Diagnoses were categorized as “Musculoskeletal” (ICPC-2 L), “Psychological” (ICPC-2 P), or “Other” (containing all other diagnoses).

### Statistical Analysis

Latent class analysis (LCA) was used to identify classes of sick-listed individuals based on their scoring on the prognostic RTW factors. LCA attempts to identify subgroups, or classes, of individuals who share common characteristics and are as distinct as possible from the other identified subgroups [45]. LCA is a cluster analysis method that has some advantages over traditional techniques. For example, LCA can produce statistical information about model fit that can help guide model selection [46]. The method is also flexible and can be used with different types of data, allows for different subgroup distributions (i.e., shape, size, and orientation), and handles missing values well [46, 47].

**Fig. 1:**
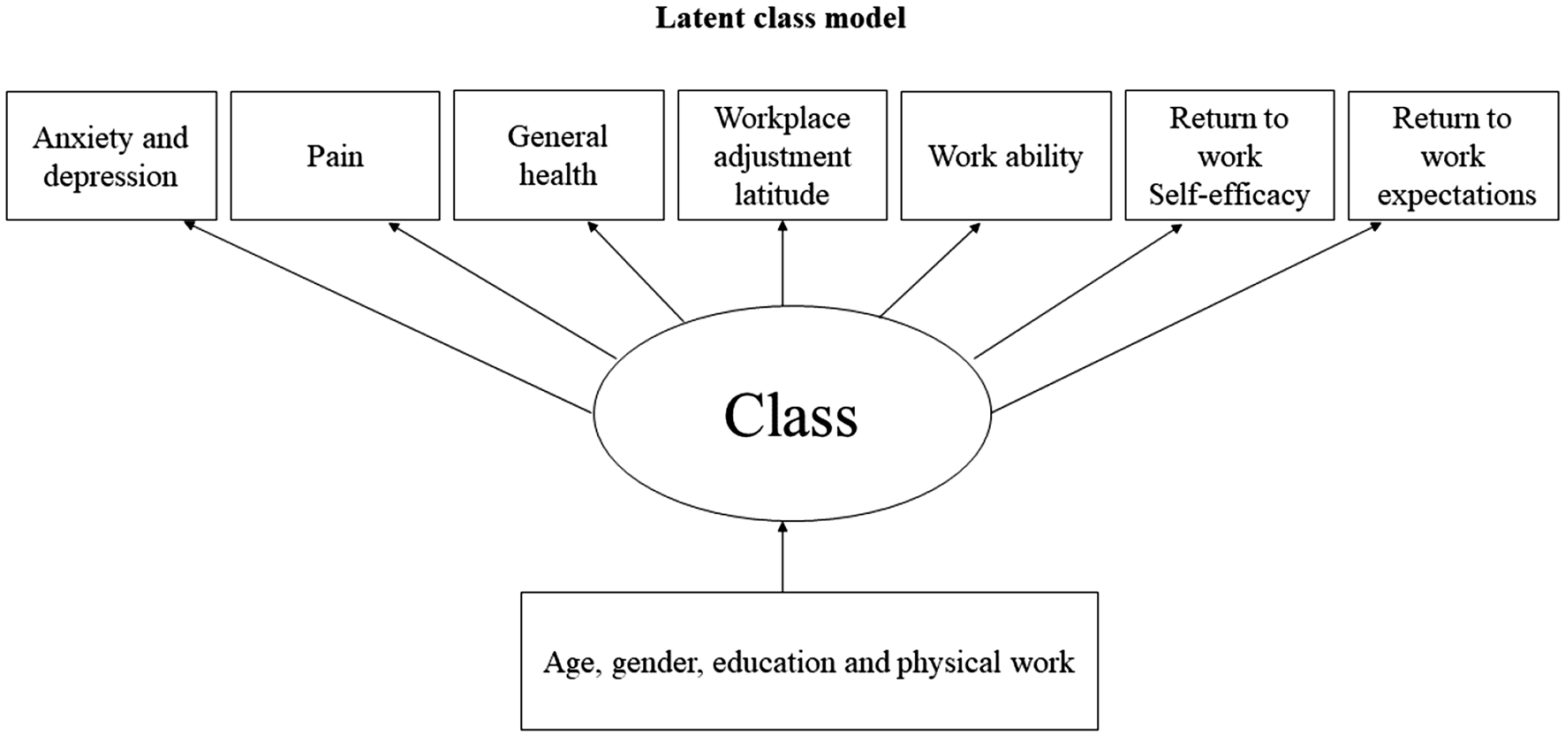
Latent class model. Indicator variables (anxiety and depression, pain, health, workplace adjustment latitude, work ability, return to work self-efficacy, and return to work expectations) and covariates (age, gender, education and physically demanding work)

The seven a priori chosen prognostic factors included as indicators in the LCA model were anxiety and depression, pain, general health, work ability, workplace adjustment latitude, return to work self-efficacy, and return to work expectations. The sociodemographic variables age, gender, educational level and physically demanding work were included as active covariates in the model (see Fig. 1).

The LCA was performed using an iterative approach starting with a model with a one-class solution and continuing up to seven classes. Model fit was assessed using the adjusted Bayesian Information Criteria (aBIC) [48] as it has been shown to be the most accurate information criteria in simulation [49]. The optimal number of classes was decided based on a combination of aBIC and substantive interpretation (i.e. if the classes are distinct and have practical meaning based on the scoring on the prognostic factors). Based on the scoring patterns, the LCA estimated posterior probabilities for inclusion into each class for each individual, and the participants were assigned to the class where they had the highest posterior probability. A posterior probability above 0.7 is recommended, and 0.9 is suggested as good when assessing uncertainty of the class assignment [50].

We tested several model specifications as suggested by Masyn [51]. First, by allowing (or not allowing) class variances to be unequal across the latent classes. Secondly by relaxing the assumption of local independence by allowing (or not allowing) indicator variables to covary within a class. For the most unrestricted model we then examined the covariance matrices for the indicator residuals in each class and identified pairs of variables whose residuals were significantly associated within a class (*p* < 0.05), indicating local dependence [52]. We then relaxed the assumption of local independence only where local dependence was indicated. For each model, a minimum of 200 random draws were performed in order to achieve an optimal model. After latent class modelling had been performed, we also examined the prevalence of the diagnosis categories within each class.

All analyses were performed using Stata 15.1 (StataCorp. 2017. Stata Statistical Software: Release 15. College Station, TX: StataCorp LLC).

## Results

### Sample Description

The final sample (*n* = 532) consisted of 65% women, 65% had higher education and the mean age was 44 years (SD 9.8). The mean symptom scores indicated mild anxiety and depressive symptoms (15.8 SD 10.2) [35], and mild to moderate pain intensity (4.3 SD 2.7) [53]. The mean work ability of 3.5 (SD 2.7) can be described as “poor” [41]. Diagnoses were split by about a third for musculoskeletal (37%), psychological (32%), and all other diagnoses (31%). See Table 2 and Fig. 3 for additional characteristics.

### Model Selection

The more unrestricted models generally had better model fit compared to the more restrictive models (see Table 1). The chosen model specification which presented the best fit included class-varying variances, as well as relaxation of the assumption of local independence for those variables that were found to covary within a class (Model 5 in Table 1). The five-class model presented the lowest aBIC and this model and those with ± one class were further examined. The four and the five class models showed similar patterns, however the four-class solution was selected based on the interpretation of practical meaning and simplicity. Posterior probabilities were generated for 532 participants, meaning 39 participants had too many missing values to be classified. The average posterior probabilities of class membership in the final model were 0.90, 0.83, 0.90 and 0.88 in Classes 1–4 respectively which indicated that subjects were classified with low uncertainty.

**Table 1:** Model fit (adjusted Bayesian Information Criteria) for the latent class models

Classes	Model 1	Model 2	Model 3	Model 4	Model 5
1	19,920	19,920	19,382	19,382	19,376
2	19,536	19,360	19,285	19,075	19,039
3	19,419	19,187	19,042	19,023	18,994
4	19,200	19,090	19,011	18,974	18,917
5	19,144	18,988	19,002	18,951	18,898
6	19,122	18,950	19,009	18,959	18,908
7	19,093	18,937	N/A*	19,010	18,911

Lower fit indices indicate a better-fitting model. Model 1: Class-invariant variances, diagonal covariances between indicator variables within classes. Model 2: Class-varying variances, diagonal indicator covariances. Model 3: Class-invariant variances, unrestricted indicator covariances (*The 7-class model failed to reliably converge). Model 4: Class-varying variances, unrestricted indicator covariances. Model 5: Class-varying variances, unrestricted indicator covariances where local dependence was indicated.

### Class Prevalence and Characterization

**Fig. 2:**
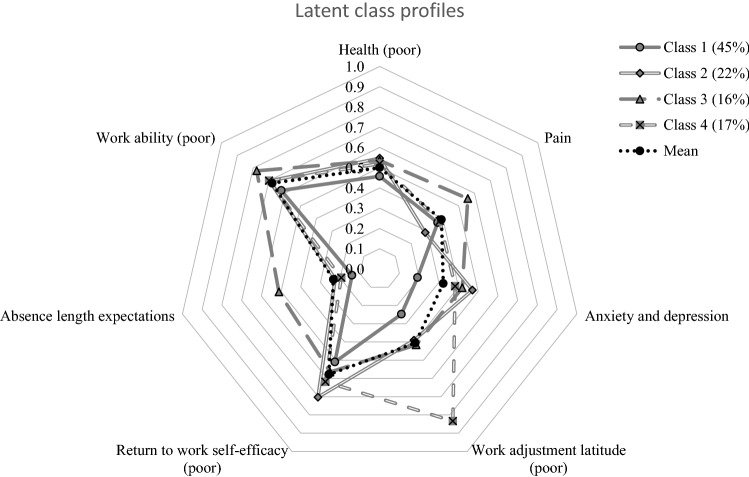
Normalized class profiles. Variables are normalized on a scale from 0 to 1, where 1 indicates poorer scores. In this representation, mean scores were divided by the variable’s full range and reversed where higher numbers originally indicated favorable scores

Table 2 describes the characteristics of the four classes and normalized class profiles can be found in Fig. 2. The first and largest class (45%, *n* = 240) was indicative of individuals who had low symptom scores, high RTW self-efficacy and high work ability. Class 2 included 22% (*n* = 114) of participants and had the highest level of anxiety and depression symptoms, poorest self-efficacy as well as younger age, less physically demanding work and higher education. The third class included 16% (*n* = 87) of participants and consisted of those with poor scores on several of the prognostic variables, including higher levels of pain, and anxiety and depressive symptoms. They also more frequently had lower education and physically demanding work. Further, individuals in Class 3 expected to be sick listed longer than the other classes. Class 4 included 17% (*n* = 91) of the participants and was characterized by moderately high pain and anxiety and depressive symptoms. Similar to Class 3, subjects in Class 4 more frequently had a physically demanding job than the sample mean, but Class 4 was also characterized by poor possibilities for workplace adjustments.

**Table 2:** Characteristics of the overall sample and classes (values given are mean (SD), unless otherwise stated)

Variable (full range)	Sample *n* = 532	Class 1 *n* = 240 (45%)	Class 2 *n* = 114 (22%)	Class 3 *n* = 87 (16%)	Class 4 *n* = 91 (17%)
Age (18–62 years)	44 (10)	46 (9)	39 (9)	45 (10)	45 (10)
Gender (female)—*n* (%)	351 (66%)	160 (67%)	81 (71%)	56 (64%)	54 (59%)
Education (higher)—*n* (%)	351 (66%)	175 (73%)	98 (86%)	31 (36%)	47 (52%)
Physically demanding work (more)—*n* (%)	179 (34%)	67 (28%)	13 (11%)	49 (56%)	50 (55%)
Self-reported health (0–100)	50.4 (20.5)	54.3 (20.9)	45.0 (18.1)	48.8 (23.3)	49.1 (18.0)
Pain intensity (0–10)	4.3 (2.7)	4.1 (2.6)	3.1 (2.6)	6.1 (2.1)	4.2 (2.5)
Anxiety and depressive symptoms (0–48)	15.8 (10.1)	9.1 (5.0)	23.8 (7.7)	20.7 (10.9)	18.5 (10.7)
Work ability (0–10)	3.5 (2.6)	4.1 (2.9)	3.4 (2.1)	2.4 (2.3)	3.4 (2.6)
Workplace adjustment latitude (1–10)	6.0 (3.0)	7.6 (2.0)	6.0 (2.2)	5.8 (3.0)	1.7 (0.7)
Return to work self-efficacy (0–5)	2.5 (1.1)	2.9 (1.0)	1.8 (0.7)	2.6 (1.1)	2.3 (1.1)
Expected sickness absence length (0–12 months)	3.0 (2.7)	1.8 (1.2)	3.0 (0.7)	6.9 (3.7)	2.2 (1.6)

Education: Percentage of individuals that have completed a minimum of 3 years of higher education at the college or university level. Physically demanding work: Percentage of individuals that rate their work as “demanding a lot of walking and lifting” or “heavy manual labour”. Self-reported health: Higher number indicate better health. Pain intensity: Higher number indicate more pain. Anxiety and depressive symptoms: Higher number indicate more symptoms. Workplace adjustment latitude: Higher number indicate greater possibility for work adjustment. Return to work self-efficacy: Higher number indicate greater self-efficacy

### Diagnosis

**Fig. 3:**
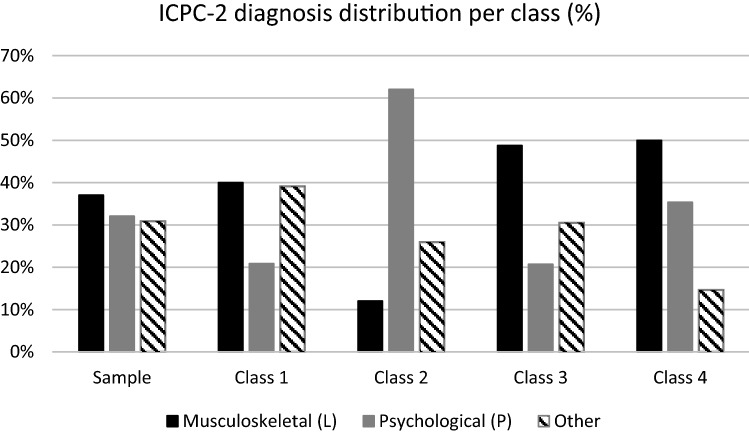
Distribution of diagnostic groups based on ICPC-2 diagnoses set by the worker’s general practitioner. Percentages within each class and in the total sample

Figure 3 describes the distribution of the diagnosis categories in the sample and classes. Participants with a psychological diagnosis was to a greater degree grouped into Class 2 (62%). Those with musculoskeletal diagnoses were more evenly distributed between Class 1 (40%), Class 3 (49%) and Class 4 (50%). Similarly, “Other” diagnoses were less frequently placed in Class 4 (15%), but more evenly distributed among the other classes. Diagnosis was missing for 30 participants in the final model.

## Discussion

This cross-sectional study identified four classes of sick-listed individuals based on seven prognostic RTW factors and four covariates. The four classes were characterized by distinct patterns across prognostic RTW factors, largely independent of sick leave diagnosis.

Previous research has attempted to define more homogeneous patient subgroups with the goal of reducing complexity and simplifying treatment options [54]. These endeavors have frequently been based on individual characteristics such as symptoms, pain sites, or other prognostic factors, and usually within defined patient groups. Previous studies have found subgroups that differ in severity [55–57], or subgroups that are qualitatively distinct, for example in symptoms or personal factors [22, 58–60]. For instance, studies using prognostic factors have found subgroups characterized by low risk, mental health issues, physical limitations and pain, and workplace related concerns in patients with musculoskeletal disorders [61, 62]. The present study adds to the previous literature by grouping individuals regardless of sick leave diagnosis into comparable subgroups that differ both in severity (i.e. most favorable scores on RTW predictors in Class 1, medium in Class 2 and Class 4, and poorest scores in Class 3) and qualitatively (e.g. mental health issues and workplace factors in Classes 2 and 4, respectively). The findings suggest that sick-listed individuals can be classified based on prognostic factors rather than diagnosis in an RTW context. A cross-disease approach in the RTW process has also previously been advocated [13].

### Implications for Practice

Identification of those at risk (or not at risk) for prolonged sick leave is important for both social insurance and vocational rehabilitation services in order to create plans for RTW [63]. This is important in order to design rehabilitation services and to allocate appropriate resources based on the expected prevalence of a risk group. Screening to identify and provide additional care to high risk groups with musculoskeletal disorders has shown to reduce time off work for these groups [24].

Class 1, with almost half of the participants, was characterized by advantageous scores on several of the prognostic RTW factors compared to the other classes. Identifying those with good prognosis may be useful in order to avoid excessive assistance (overtreatment) for these individuals [64], which may even delay RTW [65]. However, further research is needed to determine whether individuals in Class 1 have a favorable RTW prognosis.

Class 2 was characterized by younger age, anxiety and depressive symptoms, and poor RTW self-efficacy. Furthermore, Class 2 had a higher prevalence of individuals with a psychological diagnosis compared to the other classes. These characteristics indicate that work-focused cognitive therapies that could help promote self-efficacy could be useful for such a group [66, 67]. However, the mean scores for anxiety and depressive symptoms were similar in Class 3 and Class 4. This indicate that anxiety and depression symptoms were common for those with poorer prognostic scores (Classes 2–4) in the present study regardless of the prevalence of psychological diagnoses in the classes.

Class 3 was characterized by individuals who generally scored poorly on many of the prognostic RTW factors. Individuals in this class reported both pain and mental health symptoms, but more often had a musculoskeletal diagnosis than a psychological diagnosis. For those experiencing chronic pain, research has emphasized that psychosocial factors, such as fear-avoidance beliefs and psychological distress, are associated with poor outcomes [68]. Such factors are common in the first few months after injury [69] but may not always be identified when seeking help for physical symptoms [70]. Previous cluster analyses of musculoskeletal patients have also identified psychologically distressed subgroups [22, 60, 62, 71], which could be similar to Class 3 in the present study. Such groups may benefit from broader interventions also focusing on coping, problem solving, and other psychosocial factors [4, 72, 73].

Class 4 was characterized by physically demanding work in combination with poor possibilities of workplace adjustments. Although some work tasks are difficult to accommodate to individual employees, Class 4 may still indicate a proportion of sick-listed workers where workplace interventions could be sought in order to facilitate RTW as work adjustments are important for RTW [16, 31]. Where adjustments are difficult, interventions could address other aspects of the workplace, such as supervisor support, disability management practices, and workplace culture [71, 73]. Some of those experiencing low adjustment latitude after illness may also need help or encouragement in finding a more suitable job. Job changes can be a solution to ill health in order to avoid movement out of employment [74].

In patients with back pain, using risk factors to identify subgroups led to the development of the PRICE tool [61, 62, 71, 75]. The PRICE tool can be used to identify those with poor prognosis for RTW and also indicates where assistance should be focused (e.g., the workplace, psychological coping, physical activation) for this patient population [75]. The present study indicated similar subgroups independent of diagnoses as those described in the aforementioned studies, which supports the relevance of our subgrouping approach. The identified subgroups may indicate typical barriers to RTW at this stage of sick leave. Such groups could be used, for instance by social insurance workers who serve a diagnostically varied user group, as a starting point to identify problem areas that could be the focus for vocational rehabilitation interventions. For the present findings, however, further research is needed to examine the practical relevance of prognostic subgrouping across diagnoses. Identification of subgroups based on risk of prolonged sick leave may be useful in itself if the predictive validity of the classes is acceptable. However, it does not necessarily follow that such subgroups respond to interventions. Matching interventions to prognostic risk factors can be difficult and has previously been found to be lacking in practice [73]. Furthermore, investigations of intervention effects for such subgroups require separate carefully designed studies [76].

### Strengths and Limitations

Use of LCA reduced complexity of the variable combinations into four distinct groups and allowed us to identify differences between the classes of long-term sick-listed individuals across diagnoses. Further, using prognostic factors on their continuous scale in the LCA retained all information on the variables, which is useful as sick individuals usually differ on a continuum rather than by dichotomous symptom or diseases states [9]. The classes in the present study were based on a priori identified prognostic factors that are predictors for RTW, thereby increasing theoretical validity.

There are some limitations to drawing strong conclusions from this study. First, the findings in the present study needs to be replicated and the classes validated in representative samples. Additional research should also be performed with additional or different prognostic variables to examine if the class structures and prevalences are significantly altered. Second, sick leave outcomes for these classes should be investigated to examine whether the classes predict prolonged sickness absence. Finally, the study may suffer from selection bias of participants that may affect the composition and prevalence of the classes.

## Conclusions

The present study show that a heterogeneous sample of long-term sick-listed individuals can be classified into four distinct classes based on prognostic RTW factors, largely independent of medical diagnosis. These four classes differed both in severity and qualitatively across prognostic factors for RTW. Identifying subgroups based on prognostic variables might be useful to identify problem areas that could be the focus for additional RTW follow-up. Further research is needed to validate the class structure, the predictive validity of the classes and how they respond to interventions.

## Data Availability

The datasets generated and analysed during the current study are not publicly available due to protecting the anonymity of participants.
